# Glycine Signaling in the Framework of Dopamine-Glutamate Interaction and Postsynaptic Density. Implications for Treatment-Resistant Schizophrenia

**DOI:** 10.3389/fpsyt.2020.00369

**Published:** 2020-05-14

**Authors:** Andrea de Bartolomeis, Mirko Manchia, Federica Marmo, Licia Vellucci, Felice Iasevoli, Annarita Barone

**Affiliations:** ^1^ Laboratory of Molecular Psychiatry and Translational Psychiatry, Unit of Treatment Resistant Psychosis, Section of Psychiatry, Department of Neuroscience, Reproductive Science and Odontostomatology, University School of Medicine of Napoli Federico II, Naples, Italy; ^2^ Section of Psychiatry, Department of Medical Science and Public Health, University of Cagliari, Cagliari, Italy; ^3^ Department of Pharmacology, Dalhousie University, Halifax, NS, Canada

**Keywords:** N-methyl-d-aspartate, glutamate, dopamine, glycine transporter 1, PSD-95, Homer, disk-1, antipsychotics

## Abstract

Treatment-resistant schizophrenia (TRS) or suboptimal response to antipsychotics affects almost 30% of schizophrenia (SCZ) patients, and it is a relevant clinical issue with significant impact on the functional outcome and on the global burden of disease. Among putative novel treatments, glycine-centered therapeutics (i.e. sarcosine, glycine itself, D-Serine, and bitopertin) have been proposed, based on a strong preclinical rationale with, however, mixed clinical results. Therefore, a better appraisal of glycine interaction with the other major players of SCZ pathophysiology and specifically in the framework of dopamine – glutamate interactions is warranted. New methodological approaches at cutting edge of technology and drug discovery have been applied to study the role of glycine in glutamate signaling, both at presynaptic and post-synaptic level and have been instrumental for unveiling the role of glycine in dopamine-glutamate interaction. Glycine is a non-essential amino acid that plays a critical role in both inhibitory and excitatory neurotransmission. In caudal areas of central nervous system (CNS), such as spinal cord and brainstem, glycine acts as a powerful inhibitory neurotransmitter through binding to its receptor, i.e. the Glycine Receptor (GlyR). However, glycine also works as a co-agonist of the N-Methyl-D-Aspartate receptor (NMDAR) in excitatory glutamatergic neurotransmission. Glycine concentration in the synaptic cleft is finely tuned by glycine transporters, i.e. GlyT1 and GlyT2, that regulate the neurotransmitter's reuptake, with the first considered a highly potential target for psychosis therapy. Reciprocal regulation of dopamine and glycine in forebrain, glycine modulation of glutamate, glycine signaling interaction with postsynaptic density proteins at glutamatergic synapse, and human genetics of glycinergic pathways in SCZ are tackled in order to highlight the exploitation of this neurotransmitters and related molecules in SCZ and TRS.

## Introduction: Schizophrenia and Glycine Neurotransmission

Schizophrenia (SCZ) is a chronic and debilitating severe mental disorder affecting approximately 0.3–0.7% of the population worldwide (1). It is characterized by a pleomorphic symptomatology including hallucinations, delusions (“positive symptoms”), social withdrawal, avolition and anhedonia (“negative symptoms”), and deficits in multiple executive functions (cognitive symptoms). SCZ is nowadays conceptualized at molecular level as a disorder of the synaptic plasticity (2) and of abnormal cortical-subcortical connectivity (3–5). Most of the individuals affected by SCZ develop their illness in adolescence and early adulthood with about 15% showing a chronic and unremitting clinical course (6). The long-term, if not lifelong, illness trajectory, the associated high mortality, mostly determined by the elevated rates of medical comorbidities and suicide (7), and the low levels of recovery (8), make this disease a major psychiatric disorder with a great need of significant therapeutic innovation. Furthermore, the treatment response to antipsychotics, the mainstay of SCZ treatment, remains suboptimal (9). A recent study, in which analysis of 16 randomized controlled trials (RCT) were pooled together, showed that the percentage of short-term non-response ranged from 20 to 87% depending on the threshold applied, with a non-remission rate of 67% (9). In addition, a not-negligible proportion (up to 20%) of SCZ patients who are resistant to standard antipsychotic treatment, does not respond even to clozapine (10), which is the gold standard in this scenario. In this context, the identification of clinically novel effective and safe pharmacological treatments is crucial.

The interest for the role of glycine, a co-agonist with glutamate at N-methyl-D-aspartate receptor (NMDAR) in the framework of dopamine-glutamate interactions for SCZ pathophysiology and treatment, stems from the following evidence: 1) increased dopamine release in the striatum is one of the most replicated *in vivo* findings in SCZ pathophysiology (11–14); 2) all antipsychotics block or occupy dopamine D2 receptor (D2R) with no exception (15–18); 3) dopamine release is controlled, among other mechanisms, by NMDARs modulation (19); 4) NMDAR hypofunction is believed to be one of the putative pathogenetic mechanism of the disease (19, 20); 5) glutamatergic dysfunction, moreover, has been implicated even in those cases of SCZ that are not characterized by dopamine excess in subcortical regions and are not responsive to conventional antipsychotics (21–25); 6) multiple lines of evidence indicate a reciprocal modulation of both dopamine and glutamate by glycine (26); 7) over time different pharmacological glycine-centered approaches for treatment-resistant schizophrenia (TRS) have been proposed with strong preclinical rationale but mixed clinical results (27, 28).

New methodological approaches at the cutting edge of the technology such as long-timescale molecular dynamics simulations (29, 30) and single molecule fluorescence resonance energy transfer (smFRET) (31) have unveiled the very specific details of the structural and functional mutual interaction between glycine and glutamate system at preclinical level. At clinical level novel human genetic findings and imaging genetics studies link glycine signaling to SCZ. Given the extensive evidence that glycine is deeply involved in regulating glutamatergic neurotransmission, glycine reveals itself as promising potential candidate for drug discovery and safe novel pharmacological treatments. Glycine binding site on GluN1 and GluN3A NMDAR subunits is a major determinant in regulating NMDAR delivery on cell surface and furthermore influencing significantly the NMDAR activity (32).

Here, we aim to selectively review the preclinical and clinical evidence demonstrating that glycine, as well as the components of its signaling pathway, might be suitable targets for the identification of novel treatment strategies in severe psychiatric disorder and particularly in SCZ. The following research questions have led our dissertation:

1. Which is the role of glycine in the general framework of dopamine–glutamate interaction and SCZ pathophysiology?
2. How does glycine affect dynamics of post-synaptic proteins?
3. How and to what extent can glycine neurotransmission and its interaction with NMDAR be exploited to unveil novel treatments for TRS?

First, therein we describe glycine transmission, focusing on the characteristics of its components, namely receptors and transporters and their relevance in the brain circuits relevant for SCZ clinics and pathophysiology. Then, we detail the role of glycine in regulating dopamine-glutamate interaction, as well as its involvement in SCZ molecular pathophysiology. Furthermore, we review the evidence on the associations of genes encoding for elements of the glycine pathway with SCZ. Finally, a critical appraisal of potential role of glycinergic agents in treatment of psychiatric diseases is addressed.

## Dopamine, Glutamate, Glycine, and the Pathophysiology of Schizophrenia

Traditional models of SCZ focused on dopaminergic dysfunction to explain key symptoms of the disorder. This hypothesis holds that hyperactivity of dopamine transmission is responsible for positive symptoms (1) and it was formulated in the 1960s, after the discovery of antipsychotic action of chlorpromazine (33), and further endorsed by the correlation between clinical response to antipsychotic drugs and their potency to block D_2_ receptors (34, 35). Neuroimaging studies using positron emission (PET) or single photon emission (SPECT) demonstrated that, after acute amphetamine administration, patients with SCZ showed greater levels of dopamine release in subcortical regions (particularly in the striatum) compared to healthy subjects, and displayed a transient worsening of positive symptoms (11, 36), supporting the idea that hyperfunction of dopaminergic neurons is a relevant, albeit not unique, component in SCZ pathogenesis.

Administration of phencyclidine (PCP), ketamine, and other NMDAR antagonists has been known to reproduce those thought disorders observed in SCZ, such as poverty of speech, circumstantiality, and loss of goal (37). Furthermore, NMDAR antagonists may affect widespread neuropsychological domains: working memory, response inhibition, and executive processing, resulting in cognitive symptoms that are also described in SCZ, suggesting the involvement of glutamatergic neurotransmission in the pathogenetic mechanism underlying psychotic and cognitive abnormalities (38). Therefore, glutamatergic neurotransmission has been proposed as the major initial aberration in the pathophysiology of SCZ. Subcortical dopaminergic dysregulation itself might be a result of impairment in glutamatergic neurons projecting from prefrontal cortex (PFC) to midbrain dopaminergic neurons, therefore exerting control on their firing (19, 39). Indeed, in animals and humans, it has been demonstrated that NMDAR antagonist administration results in an increase of amphetamine-induced dopamine release (19, 40). These data support the hypothesis of a deficiency of glutamatergic control on dopamine neuronal activity that might underlie the increase in amphetamine-induced dopamine release.

The activation of GABAergic interneurons by glutamatergic projections is mediated by NMDAR, and NMDAR-hypofunction may specifically affect corticolimbic GABAergic parvalbumin-positive (PV+) interneurons, reducing their excitability and expression of specific molecular markers such as somatostatin and vasoactive intestinal peptide (VIP), as well as increasing oxidative stress (41). Transgenic mice with selective NMDAR deletion in cortical and hippocampal GABAergic interneurons showed specific SCZ-like phenotypes (42), supporting the so-called “GABAergic origin hypothesis” of SCZ (41).

Since NMDAR dysfunctions account for both dopaminergic and GABAergic dysregulation, it can be assumed that NMDAR dysfunction could represent the final common pathway leading from pathogenesis to symptoms (43). Glycine is deeply involved in regulating the glutamatergic transmission, acting as a co-agonist of NMDAR, allowing for its activation and enhancing excitatory glutamatergic tone (44, 45). Glycine is also involved in the regulation of dopamine transmission, exerting a multimodal action depending on its concentration and possibly inhibiting dopamine release in the striatum when administered at high doses (presumably by modulating the dopaminergic hyperfunction associated to SCZ) (46).

Potential implication of glycine signaling in the pathophysiology of SCZ is supported by a number of recent studies exploring genetic abnormalities within glycinergic system associated with SCZ as well as by the evidence of potential pro-cognitive and antipsychotic phenotype exhibited by animal models of Glycine Transporter type 1 (GlyT1) functional inhibition both by recombinant knock out (47) and pharmacological treatments (48, 49). Finally, the recent finding of elevated brain glycine and glutamate levels in patients with first-episode psychosis, measured *in vivo* by means of echo time–averaged proton magnetic resonance spectroscopy (MRS) at 4 Tesla, further confirm the relevant role of glycine in the framework of multiple interacting neurotransmitters in SCZ pathophysiology (50).

## Glycine: Functional Anatomy Relevant for Dopamine-Glutamate Interplay

### Histological Distribution of Glycinergic Neurons and Glycine Receptor

Glycine is widely distributed in the mammalian central nervous system (CNS), functioning as an inhibitory or excitatory neurotransmitter, depending on its localization. Glycine is the main neurotransmitter in inhibitory interneurons of the spinal cord, brainstem, and in some other brain regions involved in the processing of sensorimotor information and locomotor behavior (51). In the CNS, glycine is synthesized through the catalysis of serine by the isoenzyme serine hydroxymethyltransferase (SHMT), and it is largely degraded by the glycine cleavage system, also known as glycine decarboxylase complex (GDC) (52). Glycine is released by Renshaw interneurons and regulates motoneurons' excitability, exerting negative feedback through recurrent inhibition (53). Glycinergic inhibitory interneurons are involved also in the spinal reflex coordination, mediating reciprocal inhibition in stretch reflex circuits and regulating the coordination of opposing muscles (54). The anatomical distribution of glycine immunoreactive (IR) cell bodies points to the cochlear nuclei, the superior olivary complex, the medial nuclei of the trapezoid body, the cerebellar cortex, the deep cerebellar nuclei, the area postrema, and the thalamus of adult rats as main localizations (55, 56). Moreover, glycine-IR fibers are localized in the hypothalamus and basal forebrain, distant from their glycine IR cell bodies (56). Glycine receptors (GlyR) have been found enriched in the spinal cord, in apical dendrites of pyramidal neurons in the cerebral cortex (57), in the limbic system, and in the hippocampus of humans and rats (58), where they are involved in synaptic plasticity (59) and in a variety of physiological processes, especially in mediating inhibitory neurotransmission.

### Structure and Function of Glycine Receptors

Glycine can activate two classes of distinct ligand-gated ion channels: chloride-permeable inhibitory GlyRs, and cation selective excitatory NMDARs. GlyRs are ligand-gated anionic channels and belong to the pentameric Cys-loop receptor superfamily (60). Electrophysiological, immunocytochemical, and *in situ* hybridization studies have shown that GlyRs are prominent in the brainstem and spinal cord (61, 62) and detectable also in the following brain regions: prefrontal cortex, hippocampus, amygdala, hypothalamus, cerebellum, nucleus accumbens, ventral tegmental area, and substantia nigra (63–65). GlyRs exist either in homomeric or heteromeric forms and are composed by five subunits arranged symmetrically in a ring around a central Cl^−^ permeable pore. Heteromeric GlyRs are localized at the synapses and consist of three α and two β subunits, forming a pentameric receptor complex. The homomeric forms are composed of five α subunits and are located extra-synaptically. The β subunits colocalize with receptor-associated protein gephyrin, that anchors the GlyR complex at the synaptic locus, thus providing a cluster of heteroligomeric GlyRs within synapses (66, 67). The expression of α subunits changes during neurodevelopment and it is regionally specific, whereas β subunits are transcribed in all developmental stages in several regions. Recent studies have detected functional GlyRs even in absence of the glycinergic terminals in dopaminergic neurons of the juvenile immature *substantia nigra pars compacta* and in developing cortical neurons, but the function of these non-synaptic GlyRs remains unclear (68, 69). Overall, a variety of functions may be performed by GlyR, depending on the major subunit of the receptor and its oligomerization. Moreover, the pattern of GlyR expression seems to be relevant during critical stages of brain development in cortical and subcortical brain regions that have attracted the attention for the animal modeling of SCZ pathophysiology.

As an excitatory neurotransmitter, glycine acts as a co-agonist of NMDAR, allowing for depolarization, removal of the magnesium blockade and Na^+^/Ca2^+^ passage through the channel, which ultimately enhances the glutamatergic excitatory tone that is critical for learning and neuronal plasticity (44, 45). While glutamate binds to a bi-lobulated cavity in NMDAR GluN2 subunit, glycine binds to a cavity located in GluN1 or GluN3, the so-called glycine-B site or strychnine-insensitive receptor (51). Glycine may be released at excitatory sites from at least two different sources: i.e., neuronal cells *via* alanine–serine–cysteine transporter-1 (Asc-1) (70) and astroglial cells *via* the functional reversal of GlyT1 (26, 71, 72). Moreover, since it colocalizes with NMDAR at post-synaptic level (73, 74), GlyT1 is believed to modulate the excitability of NMDAR by reducing glycine levels in the synaptic cleft, thus preventing saturation of the glycine-B site (75–78).

Noteworthy, the affinity of glycine for NMDARs is significantly higher than that of GlyRs (EC50 = 134 nM *vs*. EC_50_ = 270 μm) (63, 79), thus, under physiological conditions endogenous glycine may exert mainly an excitatory effect in the hippocampus, where both GlyRs and NMDARs are expressed. On the other hand, excessive glycine produced in pathological conditions, such as ischemia and epilepsy (80, 81), may spillover into extra-synaptic sites to activate inhibitory GlyRs in order to counteract the excitotoxic damage. In these conditions, GlyR-mediated inhibitory activity may be stronger than NMDAR-mediated excitatory one, resulting in a net effect of depression of excitatory post-synaptic currents (EPSCs) (82). Therefore, levels of glycine could be the major determinants in setting the polarity of glycine's role either in brain damage, either in correcting unwanted synaptic plasticity (82, 83)

### Beyond Glycine: Other Agonists at the Glycine B-Site?

Multiple lines of evidence suggest a relevant crosstalk between glycine and D-amino acids during the neurodevelopmental stages that are critical to SCZ pathophysiology. D-Serine is synthetized in the neurons starting from astrocytic L-serine by serine racemase (SR), according to the “serine shuttle” hypothesis formulated by Wolosker, (84) and its levels in the synaptic cleft are controlled by Asc-1 transporter (70, 85, 86). Among D-amino acids, D-serine seems to be a crucial player in synaptic plasticity, such as long term potentiation (LTP) (87–91), and it has been considered the putative endogenous ligand at NMDAR glycine B-site (92, 93), since it appears to be functionally up to 100-fold more effective than glycine at potentiating NMDAR activity. The role of D-serine in the activation of NMDAR is confirmed by the reduction of synaptic transmission by treatment with D-amino acid oxidase (DAAO), which depletes endogenous D-serine but not glycine (93). Noteworthy, NMDAR responses do not seem to be fully reversed by DAAO and a “DAAO-insensitive fraction” has been shown in rat hippocampus, that accounts up to 30–50% of receptor activity (93), presumably because the remainder of the sites may be already occupied by glycine, which therefore may act in some parts of the brain and at certain stages of the neurodevelopment as the major ligand. Immunohistochemical studies comparing D-serine, glycine, and NMDARs pattern of distribution in rat brain, showed that D-serine and NMDARs overlap each other and have the highest concentration in telencephalon and developing cerebellum; conversely, glycine immunoreactivity does not correspond to NMDARs localization (except in the brainstem, where it parallels the distribution of NMDARs) but seems to prevail over D-serine in the adult cerebellum, hindbrain, and olfactory bulb (94). Papouin and colleagues performed an electrophysiological study, in order to assess the specific contribution of glycine and D-serine at synaptic and extra-synaptic NMDAR sites in CA1 region of hippocampus. Using specific enzymes that degrade either D-serine or glycine, they provide supporting evidence for assuming that D-serine may be the co-agonist at synaptic receptors, whereas glycine may act at extra-synaptic NMDARs, which have little or no role in synaptic plasticity (90). Nonetheless, Yan Li and colleagues proposed that the identity of the endogenous ligand might be determined by the level of synaptic activity, thus emphasizing the contribution of glycine in LTP induction process (91), and extending previous *in vitro* reports supporting the involvement of glycine in LTP enhancement (95). They showed that tonic activation of NMDARs in the amygdala under resting conditions may be achieved by D-serine, whereas glycine may be released from astrocytes in response to afferent impulses. Therefore, ambient D-serine may act as major ligand in absence of evoked synaptic events, while activity-dependent release of glycine may be involved in LTP-related NMDAR activation in the context of fear conditioning pathways (91). Rosenberg et al. also proposed that D-serine would not be the sole co-agonist at synaptic NMDAR sites: glycine and D-serine may have partial overlapping roles in regulating synaptic activity at NMDARs, and specific glycine effects may be revealed by deleting serine racemase (SR), the enzyme that synthesized D-serine (70). In fact, in an electrophysiological experiment, they demonstrated that the synaptic NMDAR responses were essentially unaltered in adult SR-KO mice (70). Moreover, it has been demonstrated that even the GlyT1 inhibitor bitopertin increases the magnitude of LTP in rat hippocampal CA1 pyramidal cells, and this effect likely results from an increase in the extracellular levels of glycine (96). Direct application of glycine seems to exert the same effects on LTP induction, as well as increases the amplitude of NMDAR currents of approximately 50% (96). Nonetheless, it has been reported that application of high concentrations of glycine, exceeding the synaptic concentration of the endogenous Glycine B-site agonist, produce opposite effects on NMDAR currents amplitude and LTP, consistent with the internalization of a percentage of NMDARs primed by glycine (96). Glycine and glycine inhibitors may therefore display an inverted U-shape concentration-response profile on LTP induction, for whom higher glycine B-site occupancies may lead to a lack of efficacy (49). However, taken together, these findings suggest that two endogenous co-agonists, namely glycine and D-serine, may regulate distinct populations of NMDARs, with one or the other prevailing at a given synapse, finely tuning excitatory transmission in order to diversify a wide ranging repertoire of biological effects (97).

Interestingly, several lines of evidence disclose the role of D-serine for inflammation, excitotoxicity, and epileptogenesis. Inflammatory factors (such as amyloid β and lipopolysaccharide) stimulates the astrocytes and microglia to express SR (98, 99), thus these cells become the primary source of D-serine in inflammatory conditions (100). The amount of D-serine obtained by this way promotes excitotoxic damage and synaptic dysfunction through the activation of NR2 subunit at extra-synaptic sites (101). Transgenic mouse models for amyotrophic lateral sclerosis (ALS) exhibit several-fold higher levels of D-serine in spinal cord, and the elevation positively correlates with disease progression (102). Moreover, D-serine increase in spinal cord was observed even in sporadic postmortem human ALS cases or ALS relatives (102). Furthermore, it may be of interest that SR knockout mice were protected against cerebral ischemia and excitotoxic damage (103). These findings suggest that D-serine, rather than glycine, may be a key determinant for NMDAR-mediated neurotoxicity.

Correlation between D-serine levels and SCZ is demonstrated by multiple studies (104–107) that showed the decrease of its levels in CSF and serum of schizophrenic patients. Despite convergent lines of evidence pointing to the potential of D-serine in treating SCZ, it displays a low oral bioavailability, being largely metabolized by DAAO (104). Past clinical trials have demonstrated benefits of adding D-serine to the antipsychotic therapy in SCZ and bipolar disorder (108), but these results were not unequivocally replicated (109–111), leaving aside the fact that the high doses required may provoke peripheral neuropathies and nephrotoxic effects (112–114). Rather than therapeutic agent for SCZ symptoms, D-serine has recently been proposed as promising biomarker to antidepressant response to ketamine (112), with low plasma levels of D-serine predicting ketamine efficacy.

In summary, D-serine action at NMDAR may be more relevant than originally thought, and may have a pivotal role for synaptic plasticity and cognitive functions, as well as neurodegeneration and excitotoxicity.

Finally, even if it is not the topic of this review, the role of other D-amino acids in NMDAR modulation should be acknowledged, and among all the one of D-aspartate. This amino acid has been implicated in brain development (115, 116), a feature that is specifically appealing for SCZ, that is conceptualized as a pathology of the neurodevelopment with abnormal synaptic pathophysiology and altered brain connectivity. Moreover, multiple lines of evidence from animal modeling (117, 118) to postmortem brain abnormal gene expression and epigenetics (119) indicate a potential role of D-aspartate in SCZ pathophysiology, and lay the foundation for a potential use of D-aspartate as adjunctive therapy in those cases poorly responding to conventional antipsychotics (120).

### Glycine and Neurodevelopment

Several recent studies have focused on changes in glycinergic signaling and expression pattern of glycinergic markers, such as glycine transporters and glycine receptors during the development (68, 121) making the neurotransmitter of interest for a disease believed to be of putative neurodevelopmental origin, such as SCZ. It can be hypothesized that the synaptic release of glycine is involved in the proper development of many motor and sensory circuits (i.e., auditory, visual, respiratory, and nociceptive) (122, 123). GlyRs expression is specifically regulated in terms of subunit composition during the development and throughout the CNS. Homomeric α_2_ subunits are mainly expressed during the fetal period. Thus, a developmental switch from α_2_ homomeric GlyRs to α_1_β heteromeric GlyRs takes place between the birth and the third postnatal week in rats (69). Indeed, several studies demonstrated that α_1_ is the most abundantly expressed subunit in adult rats and, since β subunit interaction with gephyrin is essential for the clustering of GlyRs in the synapses, it is plausible that the α_1_β heteromeric form of GlyRs is the most common subtype within synapses (124).

Different subtypes of GlyRs might fulfill opposite roles during the development. Since intracellular chloride concentrations are high in embryonic neurons, homomeric α_2_ GlyRs expressed during the fetal period might be excitatory, mediating the depolarizing chloride flux and the subsequent inward calcium flux. Therefore, GlyR activation may exert an excitatory action in immature neurons, whereas it mediates inhibitory neurotransmission in adult CNS, by increasing Cl^-^ permeability and leading to a membranal hyperpolarization (125). Precisely this latter subtype, α_2_ GlyR, seems to be involved in pathophysiology of the autism spectrum disorder (ASD), condition that shares many clinical features and biomarkers with SCZ (126). In ASD is assumed to be an imbalance between glutamate and glycine in favor of an increased activity of glutamatergic neurotransmission early in neuromotor development. As highlighted by genetic and functional studies, suggesting the potential role of α_2_ GlyRs in synaptic plasticity, as well as learning and memory, glycinergic signaling might be linked to social and cognitive abnormalities in ASD (127). In summary, glycine neurotransmission seems to be highly modulated during brain development. This is in line with a potential crucial involvement of this neurotransmitter in pathophysiology of a disease, such as SCZ, strongly associated to a neurodevelopmental dysregulation.

## Glycine Reciprocal Regulation of Glutamate Neurotransmission

NMDARs have unique functional characteristics: voltage dependence, calcium permeability, slow kinetics, and complex modulatory processes (128). NMDAR alone requires for an efficient gating the binding of both glutamate and a co-agonist, identified as glycine in the 1987 by Johnson and Asher (129). NMDARs are hetero-oligomeric proteins composed by a combination of different subunits called GluN1, GluN2, and GluN3. Notably, while GluN1 subunit is mandatory, different subunit composition leads to different receptor properties. The GluN1 subunit forms the glycine binding site, whereas the GluN2 subunit provides part of the glutamate binding site; moreover, the two sites appear to be allosterically coupled (130). Mice that express reduced levels of GluN1 subunits display a lowered glycine affinity and a variety of cognitive and learning defects, including hyperactivity, increased stereotyped behavior, disruptions of nest building activity, and poor performance in the Morris water maze, a measure of cued learning (131, 132). The behavioral phenotypes of these glycine-insensitive mutant mice may resemble in certain respects the positive and negative symptoms of SCZ, consistent with NMDAR hypofunction hypothesis. This evidence further emphasizes the role of glycinergic signaling in the pathophysiology of SCZ.

Glycine regulates glutamatergic neurotransmission at different levels; however, glutamate affects glycine concentration too. In fact, *in vitro* studies showed that elevated extracellular glutamate concentration reduces glycine release and high-frequency trains of stimulation decrease glycinergic inhibitory post-synaptic currents (IPSC) (133).

## Glycine Transporters 1 and 2

Glycine transporters (GlyT) are membrane-bound proteins belonging to the Na^+^/Cl^−^ dependent neurotransmitter transporters family involved in the reuptake of glycine from synaptic cleft. Two glycine transporters, encoded by different genes, are known: GlyT1 and GlyT2. They share an amino acid sequence identity of approximately 50%, but differ in their expression pattern, subcellular localization, and functional properties (125).

GlyT1 works in a bidirectional fashion with a stoichiometry of 2Na^+^/Cl^−^/Gly, regulating glycine availability in the extracellular space, and terminates glycine signaling (134), significantly modulating the glutamatergic neurotransmission. GlyT1 has long been considered as exclusively expressed by glial cells, since early immunohistochemical studies did not recognize GlyT1 neuronal forms, presumably due to epitope occlusion of neuronal protein (135). However, there is an increasing evidence that GlyT1 is also expressed in neurons throughout the brain, where it is closely associated with the glutamatergic pathway (136). In glutamatergic neurons, GlyT1 is localized in both pre-synaptic membrane and postsynaptic density, where it interacts with the scaffold protein PSD-95 (136, 137).

GlyT1 plays a pivotal role in neurodevelopment as well as in cognitive processes of adult brain, as shown by the phenotype of GlyT1 mutant mice. Homozygous GlyT1−/− mutant mice appeared normal but unexpectedly died on the first day of birth, showing severe motor-sensory deficits, suggesting a vital role for GlyT1 that, even if dispensable for embryonic development, it is crucial for postnatal survival (138). Heterozygous GlyT1+/− mice, on the other hand, survive and show promnesic phenotypes, as well as a resistance to amphetamine disruptive effect on prepulse inhibition (PPI) (47, 139, 140). PPI of the acoustic startle reflex is an operational measure of the pre-attentive filtering process known as sensorimotor gating (141, 142) that is disrupted in SCZ as well as after stimulants administration even in healthy subjects, whereas antipsychotic drugs commonly are able to reverse PPI disruption (143–145). Therefore, it has been suggested that a radical reduction in expression of GlyT1 may be responsible for sensori-motor gating deficits due to the hyperactive inhibitory glycine-mediated signaling (146), whereas a mild reduction in expression of GlyT1 might enhance NMDAR function and memory retention, and might be protective against amphetamine-induced sensorimotor gating deficits, suggesting that drugs which inhibit GlyT1 might exert procognitive and antipsychotic effects (47).

Conversely to GlyT1, GlyT2 is exclusively expressed by glycinergic neurons and localized in presynaptic terminals adjacent to the active zones. Within the glycinergic bouton, GlyT2 appears associated with the plasma membrane or as discrete clumps and it seems to be excluded from the active site of the synapse and synaptic cleft (147). Unlike GlyT1, GlyT2 is coupled to electrochemical movement of 3Na^+^, maintaining the high concentration gradient on the presynaptic terminals and refilling presynaptic vesicles with glycine (148). Further, its expression is restricted to regions with glycinergic transmission, such as the cerebellum, brainstem, and the spinal cord (135). Homozygous GlyT2 −/− knockout mice also die in the second postnatal week, however, they show a phenotype entirely different from GlyT1 knockout mice, developing a lethal motor deficiency, reminiscent of severe forms of human hyperekplexia (hereditary startle disease), characterized by muscular spasticity, tremor, and impaired motor coordination (149).

GlyT1 function is thought to be closely regulated by several molecular mechanisms, e.g., inhibition by arachidonic acid, a second messenger released following phospholipase A2 activation (150). Moreover, intracellular pH value also modulates GlyT1 activity. Low doses of Zn^++^, which is released with glutamate by different types of excitatory neurons, induce GlyT1 inhibition but have no effect on GlyT2 (151). Activation of protein kinase C (PKC), induced by sustained intracellular Ca^++^ influx, decreases GlyT1 and GlyT2 expression on the neuron surface (152). Probably PKC does not affect directly GlyTs, but intermediate substrate proteins and additional kinases such as MEK1/2 kinases or PI3-kinase and CaMKII are involved in this mechanism (152). In addition, several proteins interacting with GlyTs regulate their trafficking and recycling at the pre-synaptic terminal. Particularly, Ca^++^ influx induced by depolarization promotes the GlyT2 expression on plasma membrane surrounding the active zone and this process is thought to be regulated by the interaction between GlyT2 and syntaxin-1 (153).

In summary, it has been hypothesized that GlyTs have a pivotal role in the regulation of neurotransmission both in glycinergic and in glutamatergic synapses and several lines of research suggest that changes in the activity, density, and localization of GlyTs in glial and nerve terminals are involved in synaptic efficacy and neuronal plasticity.

## Glycine Reciprocal Regulation of Dopamine Neurotransmission

Glutamate effects on dopamine regulation have been extensively recognized, since the evidence that NMDAR-antagonists increase dopamine release in the striatum dates back to 1998. It is also well known that glycine acts as a co-agonist of NMDAR, but less is known regarding the effect of glycine on dopamine release both in cortex and in striatum. Consistent with its ability to reverse PCP-induced hyperactivity and psychotic-like symptoms, it is conceivable that glycine may decrease NMDAR-mediated dopamine release. Nevertheless, evidence in this respect is controversial and glycine seems to be able to increase, decrease, or have no effect on dopamine release.

Earlier studies on rat striatal slices unexpectedly showed a net effect of glycine to potentiate dopamine release in the striatum (154), and these results were replicated in other studies (155, 156); later this phenomenon was observed even in freely moving rats, to whom glycine was administered *via* a microdialysis probe in the anterior striatum, and who exhibited increased local release of dopamine and its metabolites (157). A recent study also shows that glycine may potentiate the excitability of dopaminergic neurons in *substantia nigra pars compacta* (SNc) by amplifying NMDAR-dependent signals. In fact, exogenous applications of glycine on midbrain slices of juvenile rats may regulate dopaminergic firing, leading to a switch from tonic spontaneous firing to the bursting activity, and then increase dopamine release at post-synaptic sites (158). If that were true, glycine effect on dopamine in the striatum would not be advantageous for treating psychosis; nevertheless, glycine could rather be involved in nigral information processing and locomotor behavior. As shown by a recent study, glycine binding site stimulants might be helpful in alleviating antipsychotic-induced EPS in treated patients rather than their psychotic symptomatology, potentially by mitigating the reduced dopamine function in the nigrostriatal pathway (159).

On the other hand, according to an *in vitro* study on mouse striatal tissue, low-dose of glycine seems to potentiate basal dopamine release from presynaptic dopamine terminals, whereas high-dose glycine showed to significantly inhibit striatal dopamine release, which would be expected to be therapeutically beneficial in SCZ (160). The same authors have later demonstrated that a GlyT1 inhibitor, ALX5311, potentiates NMDA-dependent GABA-release, and that this effect leads to significant inhibition of striatal dopamine release, supporting a model in which NMDARs have dual excitatory/inhibitory function within striatum (46). Therefore, glycine might exert an excitatory effect by acting on NMDARs located on pre-synaptic dopamine terminal or conversely, an inhibitory effect by acting on NMDARs located on GABAergic interneurons, increasing or decreasing, respectively, striatal dopamine release (46).

In summary, glycine seems to exert a multimodal effect on regulation of dopamine release, depending on the brain region in which the action is considered, glycine concentration, pre-synaptic or post-synaptic action, as well as dopaminergic functional state. This multimodal action should be taken into account to explain some inconsistent clinical effects of glycinergic agents in SCZ therapy.

Another way in which glycine modulates dopamine neurotransmission is through GlyRs. GlyRs-mediated regulation of dopaminergic firing seems to involve other neurotransmitters such as GABA (161) and acetylcholine in ventral tegmental area (162), and glutamate in nucleus accumbens (163). The role of GlyRs in the regulation of mesolimbic dopaminergic neurotransmission is confirmed by a study in which accumbal perfusion of strychnine (the GlyRs antagonist) was found to decrease dopamine levels in rats, and this effect was reverted by glycine (164). Moreover, ethanol may produce its reinforcing and dopamine-elevating effects precisely *via* GlyRs: Molander and Söderpalm showed that accumbal perfusion of strychnine decreased dopamine levels *per se*, as well as prevented further dopamine increase after ethanol administration (165). Accumbal GlyRs seem to be involved not only in dopamine elevations induced by ethanol, but also may contribute also to dopamine elevations induced by cannabinoids and nicotine (166), thus showing important implications for mechanisms related to alcoholism, other addictions and dopamine-related psychiatric disorders such as psychosis.

Glycine may affect dopaminergic output indirectly, acting on presynaptic GlyRs expressed on GABAergic terminals. Noteworthy, at birth both GABA (167) and glycine (168, 169) are excitatory neurotransmitters, but during the development, they became inhibitory ones. Ye and coauthors demonstrate that, despite these differences during the neurodevelopment, the net effect of glycine on dopamine, through GABA, in ventral tegmental area consists of a strengthening of the dopamine firing (161). Furthermore, if glycine may affect dopamine release, it is also relevant the role of dopamine in regulation of glycine release. An intriguing modality by which dopamine could regulate glycine release has been recently proposed by Shibasaki et al. (26) The authors demonstrated that dopamine may increase glycine release from cortical astrocytes by reversing the GlyT1. According to this view stimulation of dopamine receptors may change the intracellular metabolic *milieu* inducing glycolysis and oxidative phosphorylation (170), resulting in increased intracellular glycine levels. This increase in glycine concentration may reverse transport by GlyT1 (171).

Overall, in the framework of SCZ pathophysiology, a bidirectional regulation of glycine on dopamine should be conceived: in fact, changes in dopamine release could modulate glycine concentration, and in turn modify the response of NMDAR *via* Glyt1 potential reverse activity, especially at cortical level.

Therefore, an “inverse” and different mechanism from the canonical one, linking NMDAR and dopamine could be suggested in regulating dopaminergic balance, which is a crucial issue in SCZ.

## Glycine Transporters and the Postsynaptic Density

Postsynaptic density (PSD) is an electron-dense structure composed of glutamate receptors (NMDARs, AMPARs, mGluRs), proteins involved in signal transduction (disrupted in schizophrenia 1, activity-regulated cytoskeleton-associated protein, calcium/calmodulin-dependent protein kinase II, Ras GTPase, and ion channels), scaffold proteins (post-synaptic density protein 95, Shank, Homers), and cytoskeletal structures (tubulin, septin, and others) localized at the distal tip of dendritic spines at excitatory synapses (172–174). The type and the number of the proteins highly influence PSD architecture, therefore significantly impacting the synaptic plasticity and dendritic shape (172–174). PSD has been implicated in pathophysiology of psychiatric disorders, including SCZ, and their treatment (175–178).

**Figure 1:**
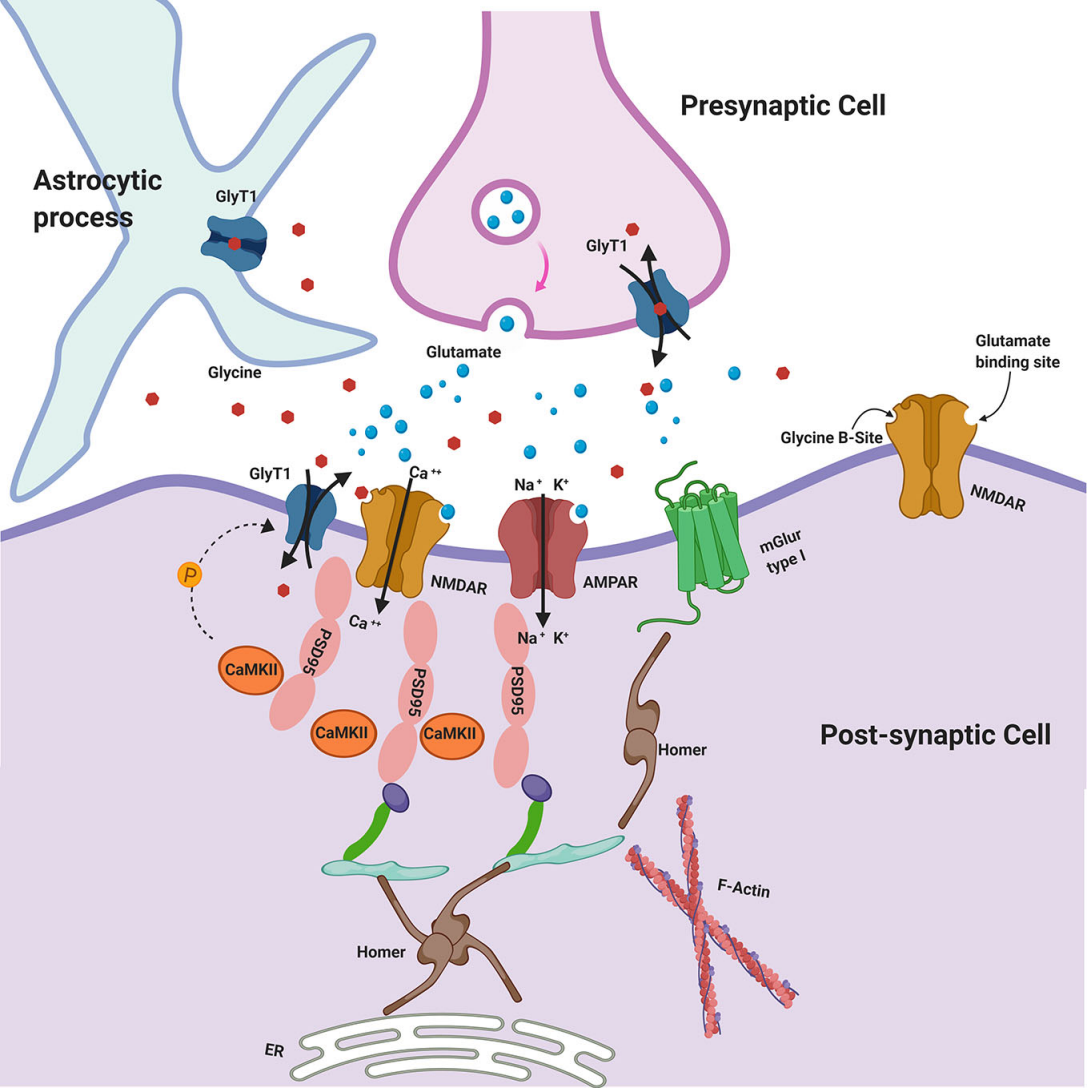
Glycine Transporters and the Postsynaptic Density (PSD). GlyT1 is localized at the PSD of asymmetric glutamatergic synapses, belonging to a protein complex including NMDAR. PSD-95 physically interacts with GlyT1, stabilizing its localization at postsynaptic membrane. CaMKII may regulate GlyT1 activity via indirect phosphorylation mechanisms. Glycine may be released in the synaptic cleft also by astroglial cells via functional reversal of GlyT1. GlyT1: Glycine Receptor Transporter 1; NMDAR: N-Methyl-D-aspartate receptors; PSD-95: postsynaptic density protein 95; CaMKII: Ca2+/calmodulin-dependent protein kinase; AMPAR: α-amino-3-hydroxy-5-methyl-4-isoxazolepropionic acid receptor; mGluR type I: metabotropic glutamate receptors type I.

In several studies postsynaptic density protein 95 (PSD-95) has been found reduced in cortical and subcortical regions of postmortem brain samples from patients affected by SCZ (179–181). Considering that PSD-95 is physically and functionally linked to NMDARs and Homer proteins (182), all believed to be involved in SCZ, it will be of interest to understand how glycine may affect and interact with PSD-95. Immunohistochemical studies showed that in hippocampus and dentate gyrus GlyT1 is localized at the PSD of asymmetric glutamatergic synapses, belonging to a protein complex including NMDAR. Therefore, recent studies have highlighted that PSD-95 physically interacts with GlyT1 in the rat brain, stabilizing its localization at post-synaptic membrane and suppressing its internalization from cell surface, thereby increasing glycine uptake (136, 137). It has been hypothesized that PSD-95 could act as a link between GlyT1 and NMDAR, regulating glycine concentration in the micro-environment of NMDAR at glutamatergic synapses (**Figure 1**) (137). In heterozygote mutant GlyT1 +/− mice, who display increased concentrations of glycine, higher levels of GluN2 and increased expression of PSD-95 have been found, suggesting that PSD-95 may anchor GluN2-containing-NMDAR at synapses (that is the NMDAR subtype mostly involved in learning and synaptic plasticity) (183), preventing their internalization (184). Other studies examined how heterozygous GlyT1+/− mice display an enriched composition of PSD, showing concomitant increased levels of GluN1/2A NMDAR subunits and GluA1/2 AMPAR subunits, since an increase in NMDARs may probably cause an elevation of synaptic AMPARs (185). Nevertheless, other recent studies have found that the AMPAR/NMDAR ratio was decreased in mutants compared to wild-type mice displaying the complexity and variability of synaptic adaptation to altered GlyT1 function (140).

Calcium/calmodulin-dependent protein kinase II (CaMKII) is a core component of the PSD that may regulate GlyT1 activity including indirect phosphorylation mechanisms (e.g., activation of a signaling cascade or the phosphorylation of cytoskeletal protein involved in trafficking of GlyT1) and there is evidence that GlyT1 is inhibited by CaMKII inhibitors (**Figure 1**) (186). Sequence analysis of SLC6 family transporters, including GlyT1, revealed multiple consensus sites for phosphorylation by kinases. It is plausible that PKCα/β also could play a regulatory role in glycine transport by phosphorylating GlyT1 (152).

The role of glycine neurotransmission in the modulation of Homer gene expression has been explored in reference to the action of antipsychotics, alone or with the adjunction of glycine B-site agonists such as D-cycloserine (187). Multiple lines of evidence suggest an involvement of Homer (long and short forms, and their splicing variants) in antipsychotics action at level of PSD (188–191). Polese et al. have shown how add-on D-cycloserine to typical (haloperidol) and atypical (clozapine) antipsychotic treatment, modulate the expression of Homer 1a, in a negative trend in caudate-putamen (187). These data and the “dominant negative” function of Homer 1a can be predictive of a relative increase of mGluR surface clustering. It could be interpreted as a mechanism of mGluR activity enhancement, mediated by NMDAR glycine site agonists.

Among proteins located at PSD, disrupted in schizophrenia 1 (DISC-1) has attracted the attention of SCZ scholars in the last decade, since human genetic, imaging genetics, and preclinical models indicate a pivotal role of this protein in SCZ pathophysiology. Several antipsychotics, as well as compounds modulating the glutamate signaling, have been demonstrated to affect disk1 expression and function. Within the scope of this review, is noteworthy that rapastinel (formerly GLYX-13), an amidated tetrapeptide (threonine-proline-proline-threonine-amide) acting as allosteric partial agonist of glycine B-site (192) has been shown to counterbalance prepulse inhibition (PPI) disruption, hyperlocomotion, and memory deficits, induced by MK-801 administration in mice as well as to revert the associated decrease in disk-1 and GluN2B proteins. It is possible that this “antipsychotic-like” effect can be mediated by GluN2B expression, since GLYX-13 seems to be ineffective in GluN2B-knockdown mice (193).

Finally, although brain derived neurotrophic factor (BDNF) cannot be strictly considered a constitutive component of PSD, it has been isolated from this structure and directly affects its function. It has been demonstrated that in rat hippocampus BDNF reduces glycine reuptake by affecting the insertion of GlyT2 in cell membrane (194). Despite the intriguing relationship between BDNF and glycine as underlined by the previous finding, the role of BDNF *in vivo* in regulating glycine pathways relevant for SCZ pathophysiology is still controversial. In fact, patients treated with sarcosine, an agonist at glycine B-site of NMDAR, display no changes in plasma levels of BDNF (195).

## Genetics of Glycinergic Pathway in Schizophrenia

A number of studies have explored the role of variation within genes encoding for elements of the glycinergic pathway in SCZ, with conflicting results in some cases. Deng et al. (196) found that a polymorphism within the SLC6A5 gene, encoding for GlyT2, was significantly associated with SCZ. Further, although indirect, support for a role of genetic variation within the glycinergic system in SCZ came from the study of Ohnuma et al. (197). These authors found that polymorphisms within the gene encoding for D-amino acid oxidase (DAO), which partially mediates the degradation of D-serine, a component of the glutamatergic transmission and an endogenous ligand for the glycine B-site on NMDAR (121), were statistically significantly associated with SCZ in a case-control study (197). More recently, a whole genome sequencing study in subjects with a high familial loading for psychotic disorders over three generations (198) found a frameshift mutation (rs10666583) in the GRIN3B gene, which codes for the GluN3B subunit of the NMDAR. This mutation was present in all family members with a psychotic disorder, but not in healthy relatives (198). The authors conclude that, given that this mutation induces an amino acid shift that degrades the S1/S2 glycine binding domain of the GluN3B subunit of the NMDAR, which subsequently affects the permeability of the channel pore to calcium ions, a decreased glycine affinity for the GluN3B subunit might cause impaired functional capability of the NMDAR (198). Of interest, a recent genetic association study in subjects with schizotypal traits showed statistically significant associations between the minor allele of three SNPs, rs2915885, rs11167557, and rs1428159, all positioned within the glycine receptor α1 subunit (GLRA1) gene, and dimensional schizotypy, specifically with the disorganized symptoms cluster (199). Further support for a role of the glycinergic system comes from a genetic-metabolomic study (200), which showed that 5-oxoproline, aspartate, and glutamate, known to affect NMDAR function, were significantly elevated in patients with rare variants in genes encoding for the glycine cleavage system. Finally, a groundbreaking study showed that genetically informed pharmacological treatment targeted at the glycinergic/glutamatergic signaling could improve significantly clinical response in patients with psychosis (201). These authors found several CNVs spanning 9p24.1 in a proband and his mother, who had diagnoses of schizoaffective disorder and bipolar disorder with psychotic features, respectively. Among the genes involved, the gene encoding for the glycine decarboxylase was of particular interest given its role in the catabolism of glycine (201). The authors performed two proof-of-principle clinical trials with glycine and d-cycloserine obtaining an additional 20 to 26% reduction in symptom severity with the former and 13 to 30% reduction with the latter (201)

Conversely, a series of negative studies do not appear to support a role for glycine transmission and signaling in SCZ molecular pathophysiology, at least on genetics ground. (202–206). The study of Feng et al. (202) explored the role of genetic mutations within the glycine receptor α2 subunit gene (GLRA2) in SCZ, using a sequencing approach. These authors detected three silent mutations in the coding region, C894T in exon 5, C1134T in exon 7, and C1476T in exon 9 (202), highlighting that a role of these variants in the pathogenesis of SCZ is unlikely. Similarly, subsequent case-controls studies confirmed these negative findings (203–206). Another negative finding derived from a gene expression analysis in *post-mortem* dorsolateral prefrontal cortex and cerebellum brain samples (207). Indeed, Burnet et al. did not find alterations of GlyT1 expression levels in these brain areas in 18 SCZ patients compared to 20 healthy controls (207).

Taken together, there is some discrepancy in the genetic findings of glycine and related signaling in SCZ. However, it should be noted that most of the early, negative, studies used case-control approaches often with inadequately powered sample sizes. The more recent translational evidence points to a role of genetic determinants in the pathophysiology of SCZ and contributes to the hypothesis that a subgroup of affected patients might take advantage of treatments targeted at the glycinergic/glutamatergic pathway.

## Critical Appraisal of Glycine Pharmacology and Its Potential Role in Schizophrenia

Several studies indicate that cognitive processes may be regulated by glycine levels at glutamatergic synapses (47, 208, 209). Glycine concentration, in turn, is regulated by GlyT1, even if the glycine-B site would be tonically saturated (210). There is consensus, however, that GlyT1 prevents saturation of the glycine binding site on NMDARs and that further glycine increase can enhance NMDAR activation (155, 211), thus representing a potential target to modulate excitatory synapses.

Recent studies in GlyT1+/− mice showing NMDAR hyperfunction, highlighted the presence of an increased number of dendritic branching in the CA1 region of the hippocampus, an enhanced synaptogenesis (184), as well as higher density of excitatory glutamatergic synapses, and an increased expression of PSD-95 compared to wild type. In summary, these results suggest that glycine contributes to the regulation of synaptic plasticity, dendritic maturation, and glutamate-induced spinogenesis in the CNS (212).

Concerning behavioral phenotype, heterozygote GlyT1 +/− mice displayed improved memory retention during spatial learning task (47), and deletion of GlyT1 in the forebrain neurons resulted in a pro-cognitive profile characterized by facilitated associative learning, working memory, reference memory, and reversal learning (208, 209). Indeed, pharmacological blockade of GlyT1 exerted pro-cognitive effects in a preclinical model of SCZ, as also showed by a GlyT1 inhibitor, PF-3463275, that has been found to reverse ketamine-induced working memory deficits (213). Furthermore, SSR-504734, another GlyT1 inhibitor, facilitated cognitive flexibility, as assessed in the attentional set-shifting task in rats (214). These compounds probably may improve cognitive function and memory by increasing NMDAR signaling (214, 215); moreover, they may increase long term potentiation (LTP) that is one of the most studied manifestations of neuroplasticity. Therefore, it might be possible that GlyT1 inhibitors can reduce psychotic symptoms by improving neuroplasticity (216).

Deletion of the GlyT1 gene causes a divergent effect on PPI, depending on regional specificity. In fact, complete GlyT1 deletion in cortex confers resistance to PPI disruption induced by the NMDAR antagonist MK-801 (217), and may lead to a “psychosis-resistant” phenotype. On the contrary, deletion of GlyT1 in the striatum provokes a relevant PPI deficit, resembling a SCZ endophenotype, and the animals remain sensitive to the PPI-disruptive effect of MK-801 (217). Hence, there is no unequivocal support to the antipsychotic potential of GlyT1 inhibition and much more remains to be discovered.

Despite the complexity of the issue, in animal models of SCZ, such as neonatal lesion of the hippocampus (218) or acute NMDAR blockade (219, 220), GlyT1 inhibitors, such as ALX-5407, sarcosine, ORG 24598, SSR504734, and SSR103800 were effective in reverting PPI disruption, thus displaying an attractive antipsychotic-like activity (217).

The therapeutic strategies based on glycine neurotransmission have yielded contrasting results with significant improvement of SCZ symptoms in some clinical trials, as well as inconclusive results or no effect at all in other studies (221) (**Table 1**).

**Table 1:** Summary of the GlyT1 and GlyT2 inhibitors and their clinical and pre-clinical effects.

Type	Compound		Mechanism of action	Functional results	References
**Sarcosine and some sarcosine-based GlyT-1 inhibitors**	***Sarcosine***		GlyT-1 inhibitor	Improvement with positive symptoms, negative symptoms, and cognitive deficits	Lane et al. (109) Lane et al. (222) Lane et al. (223)
	***NFPS/ALX5407***		GlyT-1 inhibitor	[Gly] ↑ in rodent cerebral spinal fluid (CSF), pre-frontal cortex (PFC), and cerebellum *in vivo* induction of LTP Antipsychotic and pro-cognitive effects in rodent behavioral models Enhancement of pre-pulse inhibition (PPI)	Cioffi et al. (224)
	***Org 25935***		GlyT-1 inhibitor	Reduced ketamine-induced psychomimetic and perceptual alterations in measures of total positive and negative syndrome scale	Cioffi et al. (224)
	***AM747***		GlyT-1 inhibitor	Effective as adjunctive therapy for negative symptoms in schizophrenic patients concurrently maintained on an antipsychotic treatment	Amgen et al. (225)
	***Org 24461***		GlyT-1 inhibitor		Zafra et al. (54)
	***Org 24598***		GlyT-1 inhibitor		Zafra et al. (54)
**Non-sarcosine based GlyT-1 inhibitors**	***Benzoyl is indolines***	***Bitopertin***	Selective and non-competitive GlyT-1 inhibitor	Enhancement of LTP in Sprague-Dawley rat hippocampal CA1 pyramidal neurons [Gly] ↑ in rat CSF and striatal tissues upon oral administration. Dose-dependent [Gly] ↑ in CSF in humans	Pinard et al. (226) Hofmann et al. (227)
	***Methylphenidate-derived***	***SSR504734***	GlyT-1 inhibitor	Enhancement of working memory performance in wild-type mice with high retention demand Protection against depression in the chronic mild stress model of depression Dose-dependent anti-depressant effects in rats during the Porsolt forced swim test	Singer et al. (228) Depoortere et al. (229) Boulay et al. (230)
		***SSR103800***	GlyT-1 inhibitor	Reversion of short-term memory deficit induced by phencyclidine	Boulay et al. (230)
		***GSK1018921***	GlyT-1 inhibitor	Dose-limiting AEs including dizziness and visual disturbances in humans	Cioffi et al. (224)
	***Alkyl and heteroaromatic substituted sulfonamides and sulfones***	***ACPPB***	GlyT-1 inhibitor	Promotion of dopaminergic reinnervation of the dorsal striatum; reversion of 6-OHDA-induced lateralization of sensorimotor behavior in mice	Schmitz et al. (231)
		***DCCCyB***	GlyT-1 inhibitor	Reversion of PCP-induced cognitive deficits; reversion of ketamine-induced perceptual attentional set shifting in rat models	Blackaby et al. (232)
	***Heteroaryl amides***	***PF-03463275***	GlyT-1 inhibitor	Reversion of ketamine-induced working memory deficits in non-human primates	Roberts et al. (213)
**GlyT-2 inhibitors**	***Org-25543***		Irreversible GlyT-2 inhibitor	↑ Extracellular [Gly] in the lumbar dorsal spinal cord of rats.	Whitehead et al. (233)
	***GT-0198***		GlyT-2 inhibitor	Analgesic effect in a mouse model of neuropathic pain	Omori et al. (234)
	***ALX1393***		GlyT-2 inhibitor	Inhibition of pain transmission	Morita et al. (235)

## Digging Into Potential Mechanism Responsible for GlyT1 Inhibitors Failure in Clinical Practice

To date, more than 70 placebo-controlled clinical trials of agonists or partial agonists acting at NMDAR glycine modulatory site in SCZ (including glycine, D-serine, D-cycloserine, and D-alanine) have been reported in medical literature, with controversial results (236). An alternative approach to increase glycine availability is to block glycine reuptake *via* GlyT1. However, even though several GlyT1 inhibitors seemed to be efficacious in animal models, clinical studies in humans have been disappointing at least for major endpoints. The most advanced compound tested with the highest accuracy in terms of sample size and duration of the trials, is the non-competitive GlyT1 antagonist bitopertin, which also failed to reach its endpoints in Phase III trials (236).

With respect to the failure of GlyT1 inhibitors in clinical trials, potential reasons are herein explored, in order to better understand why a promising pharmacological strategy should not be abandoned.

One aspect that could be critical in evaluating the effect of GlyT1 inhibitors, included bitopertin, is the possibility that this mechanism of action may lead to an increase in glycine levels merely at extra-synaptic sites, therefore being less effective in potentiating NMDAR synaptic function. Nonetheless, the experiments of Martina and colleagues (96), confirming the ability of bitopertin to increase the activity of synaptic NMDARs, as well as to induce LTP processes, headed in a different direction. Perhaps, bitopertin may correct a certain degree of NMDAR hypofunction, being still unable to restore completely the glutamatergic transmission. Beyond the degree of activity, NMDAR dysfunction of SCZ may lay in the decreased number of receptors (237), abnormal coupling with PSD proteins (238), altered phosphorylation status (239), and other non-neurotransmitter cues that can impact synaptic efficacy. Not all these alterations potentially occurring in SCZ can be reverted or counterbalanced merely by increasing NMDAR activity.

Moreover, we should observe that GlyT1 inhibitors display an inverted U-shape concentration-response profile of action and this element, taking into account wide inter-individual differences in drug metabolism and pharmacokinetics, may be responsible for conflicting clinical results. In fact, the inverted U-shape dose-response curve displayed by bitopertin in LTP induction processes, as well as the partial receptor occupancy needed for efficacy (<50%) (49), maybe made further complicated a successful translation from animals to patients in terms of dose-finding issues.

Another point that should be raised is that, despite the extensive preclinical evidence supporting the role of bitopertin in treating SCZ, chronic administration has never been tested in animal models, in order to exclude a potential loss of effect in prolonged treatments.

In the original protocol of the clinical study, bitopertin was co-administered with routine antipsychotic treatment (240), but the class of antipsychotic was variable within the sample. Therefore, a question remains to be answered about the possibility that a certain combination would be less effective than others, resulting in an overall lack of efficacy of bitopertin intervention. Noteworthy, preclinical studies showed that glycinergic agents, when combined with antipsychotics with different receptor profile, may exert a wide ranging molecular pattern of responses (187).

Placebo effects observed in bitopertin phase III trials were larger than in phase II, and placebo response rate was assessed at 56–61% both in DayLyte and FlashLyte studies, which could explain the failure to detect any significant difference between arms. The magnitude of symptoms improvement in the placebo groups of RCTs (even those testing antipsychotics) is considerably growing over time, as described by several reviews (241, 242), making it even more difficult for the active medication groups to separate in a statistically significant way from placebo.

Finally, it is also important to remember that bitopertin has been tested for negative symptoms of SCZ, that are not always easy to be assessed reliably, as well as are difficult to be distinguished between primary and secondary ones. Despite the validity of the assessment instruments, negative symptoms are often not a focus of assessment or treatment in clinical practice, because they are rarely responsible for acute crisis or hospitalizations (243). Noteworthy, to date, antipsychotic medications remarkably effective in treating negative symptoms are few. Trying to understand the reason of this granitic-like resistance, Velligan et al. proposed a negative symptom maintenance loop theory, wherein decreased initiation and withdrawal lead to a series of self-perpetuating outcomes (i.e., reduced responsiveness to social stimuli, low interest in relationships, and decreased reinforcements from the social context) (244). In this perspective, pharmacological treatment might struggle to break the cycle, and although they may motivate patients to increase their social drive, patients may still lack the ability to interact due to previous chronic isolation. Therefore, to achieve a tangible improvement of negative symptoms, adjunctive behavioral training may be required.

## Discussion

TRS is a major clinical and therapeutic challenge in the management of SCZ patients, representing also a crucial mental health issue for the social implications and for care costs (245, 246). Therefore, the search for new compounds alone or in combination with the available antipsychotics is warranted, especially when the gold standard (i.e., clozapine) therapy fails. Ongoing research suggests that the multidimensional symptoms of SCZ may arise from dysregulation in multiple signaling pathways that may revolve around glutamatergic neurotransmission. NMDAR may represent a converging point of environmental hits and genetic factors, leading to downstream neurochemical dysfunctions that may account for positive, negative, and cognitive symptoms. Therefore, it can be hypothesized that pharmacological augmentation of NMDAR transmission through glycine signaling enhancement might restore the function of prefrontal cortex to control dopamine release, offering a potentially useful strategy in SCZ treatment. Glycine-based treatments for SCZ have their rationale first of all for the potential of this amino acid to regulate glutamate signaling and to modulate in a reciprocal interplay dopamine release, interacting, indeed, with two neurotransmitters shown to be among the major players in SCZ pathophysiology. Moreover, it should be remarked that TRS, at least for those cases that are not fully responsive to dopamine antagonists or partial agonists, is believed to be linked to aberrant glutamatergic signaling. Several lines of evidence suggest a glutamatergic mechanism of action even for clozapine that, coincidentally, is significantly effective in TRS. This superior efficacy is presumably due to an additional mechanism to D2 receptor occupancy and possibly to a pro-glutamatergic action. Indeed, it has been hypothesized that clozapine may have an intrinsic agonist or partial agonist activity at the glycine B-site, that may contribute to its unique clinical effects (24).

It is clear that, despite the relevance of the issue and the strong neurobiological rationale, glycine-based pharmacological interventions are still inconclusive but, at the same time, strongly suggestive of the high therapeutic potential, especially for the severe form of TRS.

How the utilization of glycine enhancers or modulators can be improved for SCZ therapy? A first level of analysis should clearly separate the strategies based on potentiation of transmission at NMDAR glycine B-site from the ones based on GlyT1 inhibition. Comparing the outcomes of the two types of strategies may be important to try to figure what kind of targets are respectively achieved in terms of clinical improvement, therefore a focused (meta?) analysis is needed.

From the perspective of clinical trial methodology, some trials with compounds active at NMDAR glycine B-site have shown positive results; however, larger sample size and more homogeneous subsets of patients, separating those with prevalent positive or negative symptoms and longer duration of treatment, should be required. A number of measures should be considered in order to minimize the placebo response, including reducing the number of collaborating study sites and recruiting patients preferably from academic ones (241). A better evaluation is needed to determine which patients should be treated only with glycine-based pharmacological intervention, and in which ones these agents should be administered in augmentation with canonical antipsychotics. Finally, clinical trials using glycinergic agents are not always designed specifically for TRS patients, therefore an effort for including this class of patients should be done.

Furthermore, for more TRS “tuned” treatment based on glycine signaling, a better knowledge of the major kinetic steps responsible for the activation of glutamate-bound NMDAR by glycine is paramount to elucidate the pharmacodynamics of glycinergic compounds (247). Therefore, how ambient glycine levels regulate NMDAR function under a pattern of multiple stimulations, how glycine transporters interact with multiple PSD proteins, and how glycine affect overall dopamine–glutamate interaction are key questions for the development of new compounds.

In conclusion, despite the mismatch between the significant advance of our knowledge of glycine signaling in the modeling of SCZ pathophysiology and the results of clinical trials, glycine–based pharmacological therapy, alone or in combination with available antipsychotics is still worth to be explored and refined.

## Author Contributions

AdB conceived the project of the manuscript. AdB, FM, AB, and LV conceived the strategy for literature retrieval and selection. AdB, AB, MM, LV, and FM participated in the writing process of the first draft of the manuscript. AdB, MM, and FI revised the final version of the manuscript. All authors have read and approved the final version of the manuscript.

## Conflict of Interest

The authors declare that the research was conducted in the absence of any commercial or financial relationships that could be construed as a potential conflict of interest.
